# Preparation and Characterization of Potato Starch Copolymers with a High Natural Polymer Content for the Removal of Cu(II) and Fe(III) from Solutions

**DOI:** 10.3390/polym12112562

**Published:** 2020-10-31

**Authors:** Beata Schmidt, Joanna Rokicka, Jolanta Janik, Katarzyna Wilpiszewska

**Affiliations:** Department of Chemical Organic Technology and Polymeric Materials, Faculty of Chemical Technology and Engineering, West Pomeranian University of Technology in Szczecin, 70-322 Szczecin, Poland; Joanna.Rokicka@zut.edu.pl (J.R.); Jolanta.Janik@zut.edu.pl (J.J.); Katarzyna.Wilpiszewska@zut.edu.pl (K.W.)

**Keywords:** starch, starch copolymers, polyacrylamide copolymers, superabsorbent polymer (SAP), adsorption of metal ions

## Abstract

Cross-linked potato starch (StMBA) and starch-g-polyacrylamide materials with a high content of natural polymer from 60 to 90 wt.% (St60–St90) were synthesized by double chemical-chemical modification (grafting and cross-linking). Eco-friendly starch absorbents were tested for removal of Cu^2+^ and Fe^3+^ from aqueous solutions. The characteristics of the obtained materials (Fourier transform infrared (FTIR), differential scanning calorimetry (DSC), thermal analysis (TGA), X-Ray Diffraction (XRD) and laser scanning microscopy (LSM)) confirmed their diversity in terms of composition and structure. The effect of *N*,*N*’-Methylenebisacrylamide (MBA) and polyacrylamide (PAM) content in the starch graft copolymers, treatment time and concentration of metal ions on adsorption efficiency were investigated. The adsorption efficiency for StMBA was 14.0 mg Cu^2+^/g and 2.9 mg Fe^3+^/g, regardless of the initial concentration of ions, whereas for starch graft copolymer St60 it was 23.0 mg Cu^2+^/g and 21.2 mg Fe^3+^/g. Absorption of Fe(III) was persisted even after 2 days. Pseudo-second order model was used to investigate the adsorption mechanisms. It was found that in addition to the chemical adsorption of ions on the surface, there is sorption inside the polymer network and chelating mechanism may dominate. Satisfactory results were attributed to the adequate grafting of PAM onto starch, the ability to form complexes with metal cations and changes in material structure.

## 1. Introduction

It is known that naturally occurring starch is a renewable, cheap, biodegradable, and easily available polysaccharide obtained from plants [1,2]. Natural starch is insoluble in water at room temperature, which in most cases eliminates it from industrial non-food applications. However, due to the relatively easy modifications, it is still of interest to scientists as a substrate for new sorbents [3,4,5,6,7,8]. Starch is an environmentally friendly alternative to synthetic materials obtained from petroleum-based raw materials.

Superabsorbent polymers (SAP), as three-dimensional crosslinked hydrophilic polymers are capable of absorbing and retaining significant amounts of water, dyes, or heavy metals from wastewater. These pollutants, apart from the color of the wastewater, cause many dangerous damages to organisms (metabolic, endocrine, dermatological, and nervous system destruction) [9]. The SAPs adsorption properties are widely used, e.g., in hygienic products [10,11], slow-release fertilizers [12,13], drug delivery systems [14,15] and dewatering materials [16]. Synthetic SAP’s can cause environmental problems due to their poor biodegradability. Therefore, the synthesis of SAP from natural polymers seems to be more ecological, economical and repeatable.

Starch can complex various metal particles [17,18,19], but it is much more effective after modification with appropriate groups through esterification, grafting, cross-linking and oxidation reaction of hydroxyl groups [20,21,22,23,24,25]. Starch cross-linking is a popular method of starch modification [26,27,28]. Natural polymers with hydroxyl groups cross-link upon reaction with the cross-linking agent through at least two hydroxyl groups on one polymer molecule or adjacent chains. For this reason, bifunctional [29,30] or multifunctional [31,32] compounds are used to cross-link starch. Often double starch modifications are used to obtain the required sorption properties. The double starch modifications are physical-chemical, physical-enzymatic, chemical-enzymatic and the most widely used chemical-chemical processes [25,33,34,35,36], for example grafting and cross-linking [6,29,30,31,37]. The cross-linking of starch depends on many factors, such as the type of starch, its origin, the type of cross-linking agent and its concentration, the reaction time, the initiator used, and others [29,30,31,32,33,34,35,36,37].

The results of the absorption of heavy metals by modified starch sorbents were collected by M. Haroon et al. [25]. However, this paper lacks the results concerning Cu(II) adsorption efficiency for cross-linked starch-graft-polyacrylamide sorbents. Literature reports regarding this type of starch copolymer are often devoted to merely water capacity [29,30,38]. Moreover, the absorption of Fe(III) cations by acrylamide starch copolymer is considered in the context of water purification by flocculation [39,40]. Therefore, in the presented research, the sorption of copper(II) and iron(III) cations from aqueous solutions and swelling properties of SAPs were investigated and compered to raw potato starch and crosslinked starch. A novelty in these studies is also the use of SAPs with high starch content for the purification of cation solutions. The results of the adsorption efficiency have not been found in the available literature for identical materials. Furthermore, pseudo-second order model was adopted to investigate the adsorption mechanisms. The mechanism of sorption by the tested materials was proposed.

In this work, grafting and crosslinking modified potato starch and starch-g-polyacrylamide were synthesized. The starch SAPs were synthesized by free radical initiated polymerization with ammonium persulfate as initiator. Eco-friendly SAPs with high starch content ware characterized using Fourier transform infrared (FTIR) spectroscopy, differential scanning calorimetry (DSC), thermal analysis (TGA), X-Ray Diffraction (XRD) and laser scanning microscopy (LSM). Sorption studies of starch SAPs were carried out depending on treatment time, initial concentration of metal ions, content of cross-linker agent as well as content of polyacrylamide in the material. This manuscript partially fills “the research gap” concerning cation absorption in starch-g-polyacrylamide cross-linked materials. These results are of value for the development of “green SAP” for wide applications.

## 2. Materials and Methods

### 2.1. Materials

Potato starch (St, standard quality) with moisture content about 16.5 wt.%, average molecular weight 4.37 ∙ 10^7^ g/mol, 29.7 wt.% amylose content was purchased from Potato Industrial Enterprise “Nowamyl” S.A., Nowogard, Poland. Acrylamide (~99%, AM) and *N*,*N*’-Methylenebisacrylamide (~99%, MBA) from Merck, Germany, were used as monomer and crosslinker, respectively. Ammonium persulfate (ASP)—polymerization initiator (~99%) and acetone (analytical reagent grade) were purchased from Chempur, Piekary Slaskie, Poland. Aqueous metal salt solution Fe(III) and Cu(II) were prepared from hydrated sulfates (analytical grade, POCH, Gliwice, Poland). All materials were used without further purification.

### 2.2. Preparation of Starch-g-Polyacrylamide SAPs with High Starch Content

**Scheme 1:**
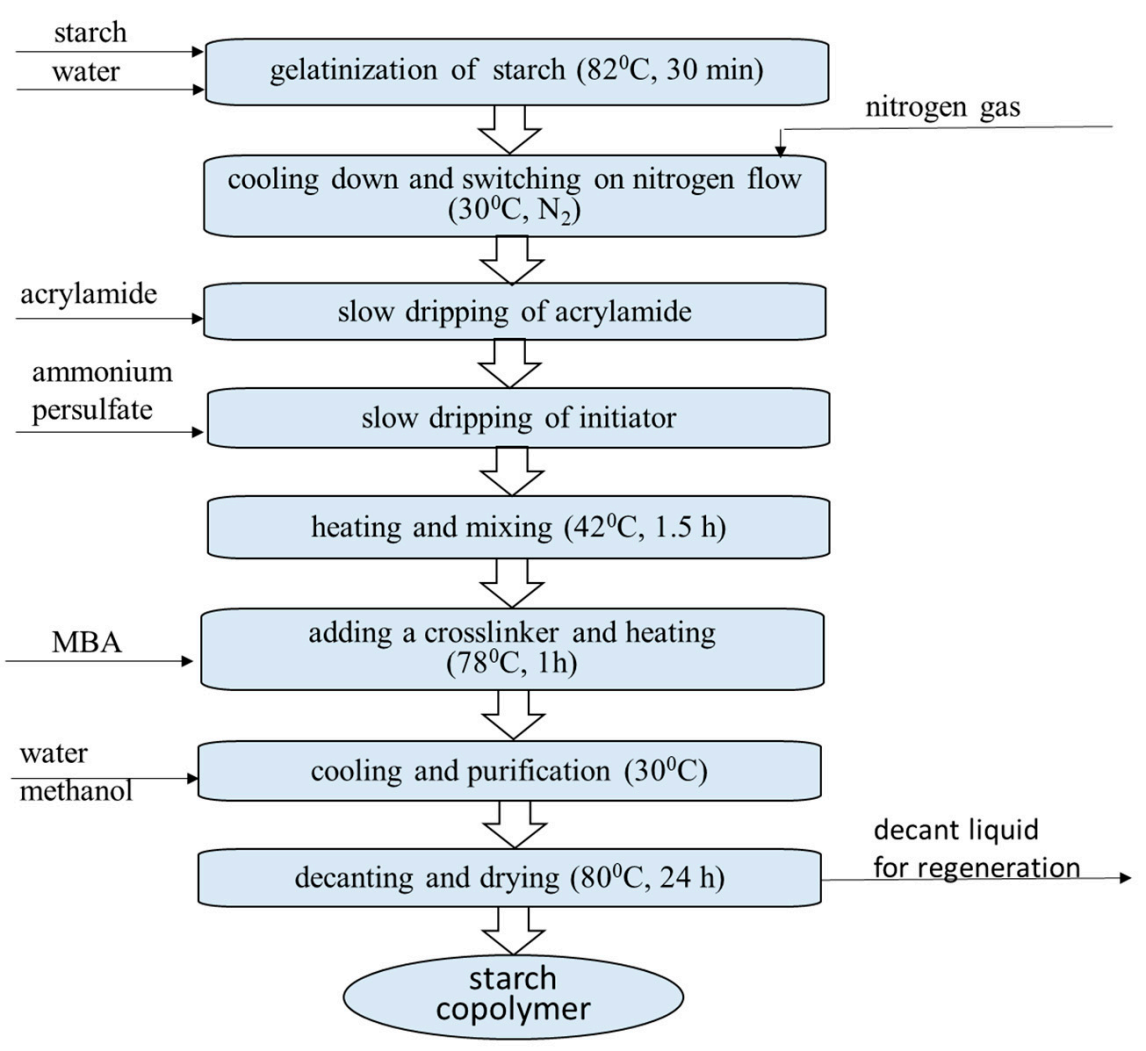
The schematic diagram of unit operations during syntheses of starch-g-polyacrylamide superabsorbents.

The cross-linked acrylamide starch copolymers were synthesized by free radical initiated polymerization of potato starch (St, 2.28 g) with acrylamide (AM content from 10 to 40 wt.% on the starch weight). Polymerization initiator ASP was used in 1.0 wt. % on the weight of AM and MBA in 0.5 wt.% or 1.0 wt.% on the substrates weight. A total amount of 75 cm^3^ of distilled water was always used in the synthesis. The detailed procedure of obtaining starch superabsorbents is presented in the form of a schematic diagram (Scheme 1). The starch was gelatinized at 82 °C for 30 min. After gelatinization, the starch was stirring under nitrogen atmosphere and cooled to 30 °C. The grafting reaction was carried out at 42 °C for 1.5 h. The cross-linking performed at 78 °C during 1 h. Obtained materials ware washed with distilled water and methanol several times to purify SAPs from the free polyacrylamide polymer. The gelatinized potato starch and starch crosslinked copolymers were dried at 80 °C for 24 h.

### 2.3. Fourier Transform Infrared (FTIR) Spectroscopy

The FTIR spectra of dried powdered samples of starch copolymers from 60 to 90 wt.% of natural polymer, potato starch and crosslinked potato starch were obtained using Nexus FTIR spectrometer (Thermo Nicolet Corp., Waltham, MA, USA) with Golden Gate (ATR) attachment and OMNIC computer program. The spectra were baseline corrected and were collected at a resolution of 0.5 cm^−1^ in the range of 500–4000 cm^−1^ for all the samples.

### 2.4. Differential Scanning Calorimetry (DSC)

Q100 DSC (TA Instruments Inc., New Castle, DE, USA) was used for the starch-g-polyacrylamide absorbents and crosslinked starch characterization (10 °C/min, from 0 to 250 °C in nitrogen atmosphere). Samples were weighed (~5 mg) on the aluminum pans and hermetically sealed before analyses.

### 2.5. Thermogravimetric Analysis (TGA)

A TGA Q5000 TA Instruments thermal analyzer was used to determine the decomposition temperature and weight loss of starch-polyacrylamide SAPs. The absorbent samples were heated from 20 °C to 900 °C with the heating rate of 10 °C/min.

### 2.6. Laser Scanning Microscopy (LSM)

The surface morphology of selected freeze-dried (Christ Alpha 1–2 LD plus Instrument with condenser temperature—<−50 °C and chamber pressure—<0.120 mbar was used; the freeze-drying time was 48 h) St90, St60 and starch samples was observed by laser scanning microscopy (LSM), model VK 9700 (Keyence, Mechelen, Belgium) equipped with a violet laser source (wavelength 408 nm) and a pin hole optical system. During LSM analysis the field of the microscope was scanned using a laser beam and an X–Y scan optical system. Application of the optical system assured high measurement accuracy. The area of the measured samples was scanned several times by moving the objective lens along the Z-axis, and the intensity of the reflective light was obtained on the basis of the Z position. The magnification of 3000 was used for the measurements. Surface images of the samples were prepared using the VK Analyzer software (Keyence, Mechelen, Belgium). The samples were freeze-dried to remove moisture and homogenize the material before microscope imaging.

### 2.7. X-ray Diffraction (XRD)

Potato starch and starch absorbents were characterized by the XRD method. X-ray powder diffraction (XRD) patterns of the samples were performed on Empyrean (Malvern Panalytical, Malvern, UK) X-ray diffractometer employing CuKα radiation (λ = 1.544 Å). The microstructures of SAPs were characterized with a generator voltage of 40 kV and a tube current of 7.5 mA. The scanning range of the diffraction angle 2θ were from 7° to 90° with a step of 0.02° and a step interval of 0.01°/s. Starch moisture was 16.5 wt.%.

### 2.8. Adsorption of Metal Ions

All sorption tests (adsorption of copper(II) and iron(III) cations from aqueous solutions together with swelling) were carried out on mechanically powdered materials separated by a set of vibrating sieves. The powder fraction with particle diameter of less than 0.3 mm was used in sorption studies.

**Figure 1:**
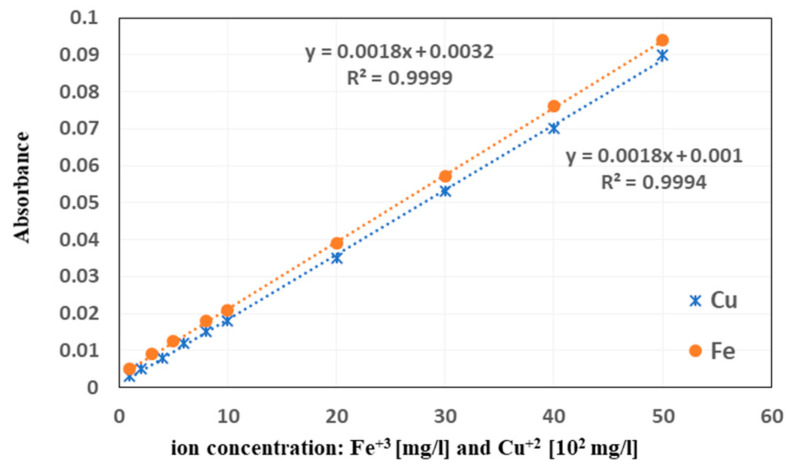
The obtained calibration curves for Fe^3+^ and Cu^2+^ ions solutions.

Cooper(II) and iron(III) cations were adsorbed from aqueous solutions with the initial concentrations of 50 and 100 mg of Cu(II) in 100 cm^3^ and 10, 30 and 50 mg of Fe(III) in 1000 cm^3^. Measurements of the changes in absorbance of colored solutions of Fe^3+^ and Cu^2+^ ions in time were determined spectrophotometrically (UV-9000 apparatus, BioSens, Ningbo, China) using wavelengths: 480 nm for Fe^3+^ solutions and 608 nm for Cu^2+^ ions. Figure 1 shows the obtained calibration curves for individual ions. Additionally, the concentrations of the investigated metal cations were controlled by atomic absorption spectroscopy (Hitachi Z-2000, Mannheim, Germany).

The ion adsorption was carried out by using 50 mg ± 2.0 mg of starch sorbents added to 50 cm^3^ of the appropriate cation solution and the resulting dispersion was maintained at room temperature (25 °C ± 1 °C) for 2 days. At that time, samples were taken for analysis, initially every minute, then every 10 min, and finally the tests were carried out after 24 and 48 h.

The removal of cations from solution (R) was calculated from the following Equation (1):

R = [(C_0_ − C_t_) · 100%]/C_0_, (%) (1)

where: C_0_ is the initial ion concentration (mg/L), C_t_ is the ion concentration after time t (mg/L) calculated from calibration—Figure 1.

The adsorption efficiency—the amount of metal ion bond by starch absorbent (mg/g)—(AE) was calculated with Equation (2):

AE = [(C_0_ − C_t_) · V]/W (2)

where: V is the volume of aqueous phase (L) and W is the amount of the dried absorbent (g).

To investigate the adsorption mechanisms, pseudo-second order mathematical model was used [41]. The integral form of the model predicts that the ratio of the time/adsorbed amount should be a linear function of time. The second order kinetics may be tested on the basis of Equation (3):

dAE_t_/dt = k(AE_e_ − AE_t_)^2^ (3)

where: AE_t_ and AE_t_ are the values of amount adsorbed per unit mass at equilibrium and at any time t and k is the second order rate coefficient. Separation of the variables followed by integration and application of the boundary conditions (AE_t_ = 0 at t = 0 and AE_t_ = AE_t_ at t = t) yields a linear expression of the form—Equation (4):

t/AE_t_ = 1/(k∙AE_e_^2^) + (1/AE_t_)∙t (4)

kinetic coefficient k often depends on the applied operating conditions, namely initial metal concentration, temperature, agitation rate, etc.

### 2.9. The Swelling Properties

The swelling measurements were performed at room temperature for samples with different starting metal ion concentration in the solution after 48 h. After the allotted time, the excess of solution was removed from the swelling samples by filtration. The swelling properties of acrylamide starch copolymers were carried out on samples weighing 50.0 mg ± 2.0 mg. The mass swelling was determined using Equation (5):

Mass swelling = [(M − M_0_) · 100%]/M_0_, (%), (5)

where: M is the mass of swollen sample and M_0_ is the mass of dried absorbent.

The swelling tests were carried out on three samples of the same material and the results are averaged.

## 3. Results and Discussion

### 3.1. The Double Starch Modifications

**Scheme 2:**
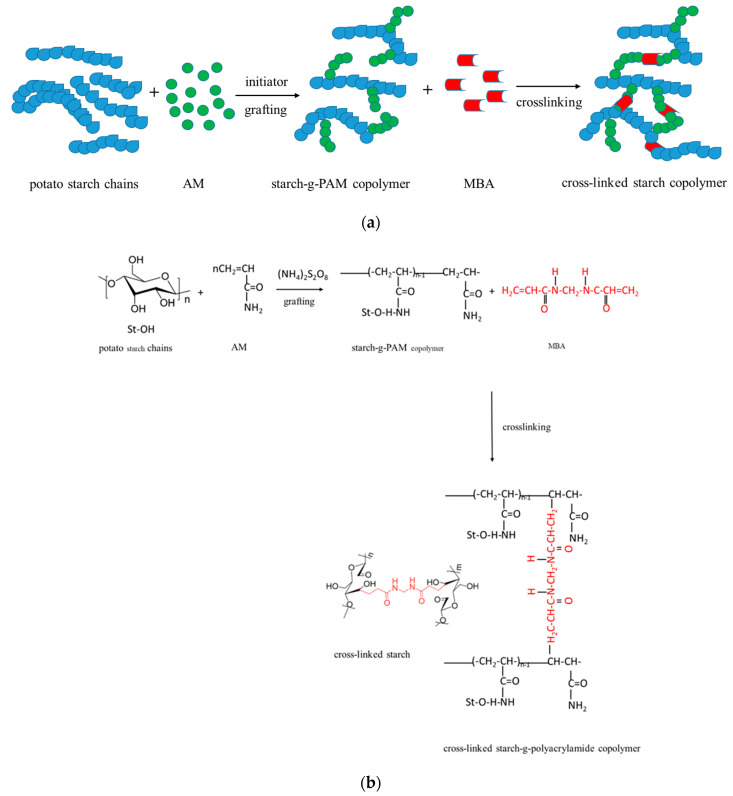
Schematic illustration: (**a**) of representation for formation of three-dimensional network of cross-linked starch-g-PAM absorbents; (**b**) of grafting starch by polyacrylamide and cross-linking starch SAP formation.

The double grafting and cross-linking starch modification was carried out by free radical initiated polymerization of potato starch (St) with AM (St90 includes 10 wt.% AM, St80—20 wt.% AM, St70—30 wt.% of AM and St60—40 wt.% AM on the starch weight). The diagram for the formation of the three-dimensional cross-linked starch-g-PAM network is shown in Scheme 2a. The Scheme 2b presented the step by step formation of cross-linked starch grafted polyacrylamide copolymers: (i) formation of a starch grafted polyacrylamide copolymer in a free radical reaction; (ii) cross-linking of the starch-g-PAM by the MBA and (iii) formation of the starch SAP materials.

The cross-linked starch—SAP presented in Scheme 2 contained three types of chains in its network, in addition to starch chains. These are: grafted polyacrylamide (PAM) chains with one end connected to –OH on anhydroglucose units, PAM chains grafted onto starch linked with the cross-linking agent MBA [30] and, due to the fact that MBA is an excellent cross-linking agent for starch [25,42], cross-linked starch chains. Therefore, for comparative purposes, cross-linked starch SAPs were also synthesized without PAM grafting at two MBA concentrations (StMBA1-0.5 wt.% and StMBA2-1.0 wt.%). Increased cross-link density and less stretched starch SAP chains increases the resistance of the gel matrix to external stresses [30] (higher thermal resistance).

### 3.2. Characterization of Modified Starch SAP

#### 3.2.1. FTIR Analysis

**Figure 2:**
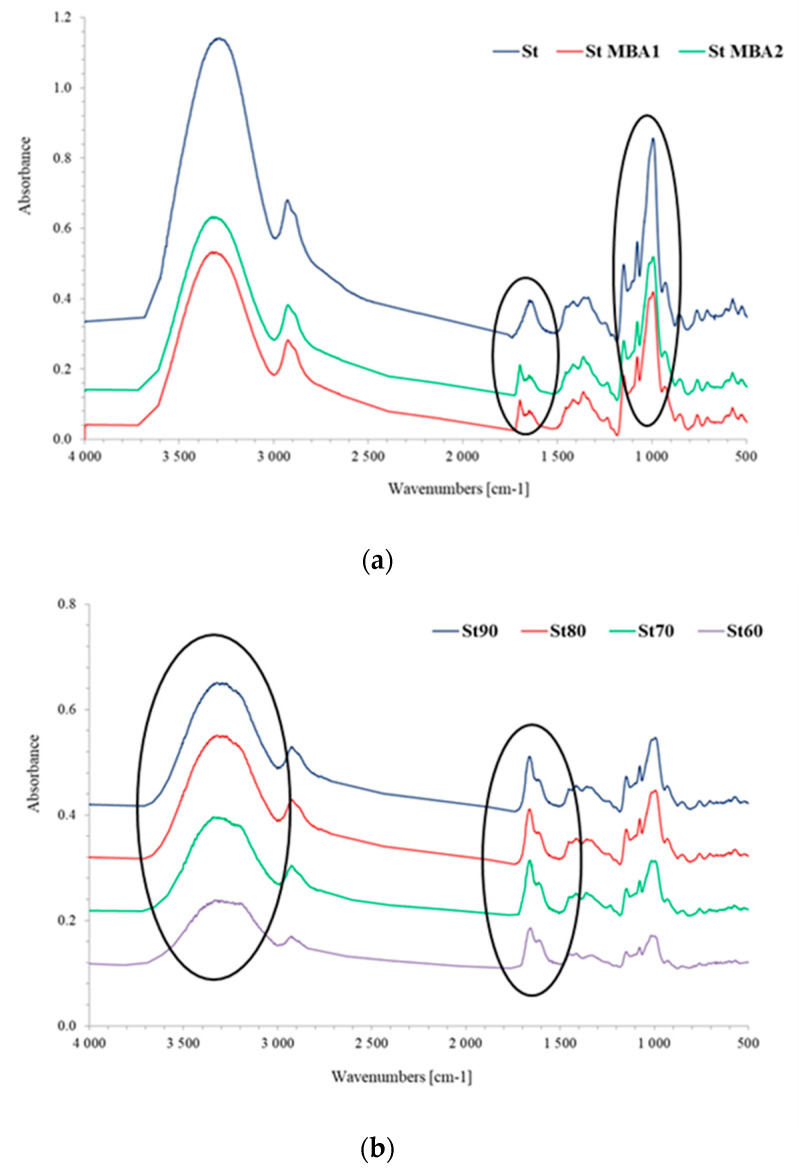
FTIR spectra: (**a**) native potato starch (St) and cross-linked starches (StMBA1 and StMBA2); (**b**) cross-linked starch-g-polyacrylamide absorbents with different amount of natural polymer St90—90 wt.%, St80—80 wt.%, St70—70 wt.% and St60—60 wt.% of starch.

The confirmation of double chemical modification of starch was made by the infrared spectroscopy. Figure 2 shows the FTIR spectra of native potato starch (St) and cross-linked starches (StMBA1 and StMBA2)—Figure 2a and cross-linked starch-g-polyacrylamide absorbents with different amount of natural polymer from 60 wt.% (St60) to 90 wt.% (St90)—Figure 2b. In the FTIR spectrum of St (Figure 2a) the absorption band at the wavelength range 3650–3000 cm^−1^ ascribes to the –OH groups stretching vibrations and at 2900 cm^−1^ to the C–H and symmetrical CH_2_ groups stretching vibrations. A typical for starch FTIR spectra, the absorption bands at 1160, 1080 and 1015 cm^−1^ are attributed to C–O–C stretching vibrations. Additionally, the absorption bands at 1460 and 1380 cm^−1^ belong to O–H in-plane bending vibrations. The peak at 1640 cm^−1^ can be ascribed to the water absorption on the starch amorphous region in the native starch [43].

The new adsorption peaks of StMBA1 and StMBA1 at 1680 cm^−1^ and 1610 cm^−1^ proved the cross-linking reaction between St and MBA [42]. The absorption bands at 1680 cm^−1^ and 1610 cm^−1^ corresponded to the stretching vibration of –C=O (amide I) and –N–H bending vibrations (amide II) [38] in cross-linked starch. Also, the adsorption bands at 1160, 1080 and especially 1015 cm^−1^ became sharp thanks of the active groups and the elongation molecular chain with the cross-linking action [42]—marked areas in Figure 2a.

In Figure 2b, the two areas confirming PAM grafting on starch are marked. As the PAM content in starch SAP increases, the absorption peaks at the wavelength range 3650–3000 cm^−1^ (stretching vibration of –OH groups and –N–H groups resulting from PAM) flatten with the shift to the lower wavenumber, which was ascribed to the cross-linking reaction of weakened hydrogen bonding interaction [42] and the presence of polyacrylamide. The St90-60 samples show three characteristic groups derived from CONH_2_: –C=O at 1680 cm^−1^, and –N–H at 1610 cm^−1^ and –C–N at 1405 cm^−1^ [38]. From the FTIR results it can be concluded that PAM was successfully grafted onto starch chains and cross-linked starch-g-PAM with high content of starch in materials was obtained.

#### 3.2.2. The Surface Morphology

**Figure 3:**
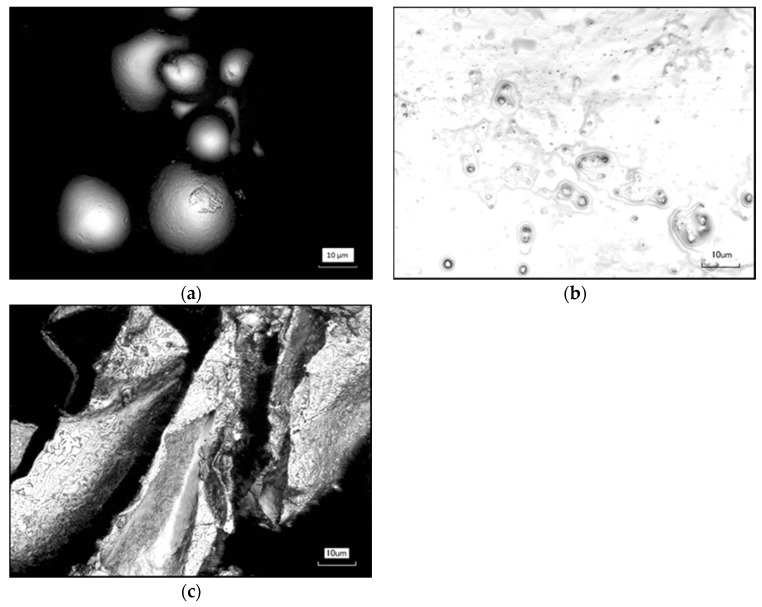
LSM of (**a**) native potato starch, (**b**) starch SAP St90 and (**c**) starch SAP St60 images.

The surface morphology of potato starch St and the starch SAP with marginal contents of St (St90 AND St60) is presented in Figure 3. Potato starch occurs in the form of irregular, circular granules of various sizes (Figure 3a). The LSM of SAP St90 (Figure 3b), containing the lowest content of grafted PAM, shows a transparent material with a rough surface with numerous PAM inserts. On the other hand, SAP St60, containing the largest amount of AM—Figure 3c, shows a lamellar structure characteristic for PAM contents in the material [44]. The St60 lamellas have a heterogeneous surface and are partially open. The images obtained by the LSM method confirm the variety of the synthesized starch SAPs.

#### 3.2.3. Differential Scanning Calorimetry

**Figure 4:**
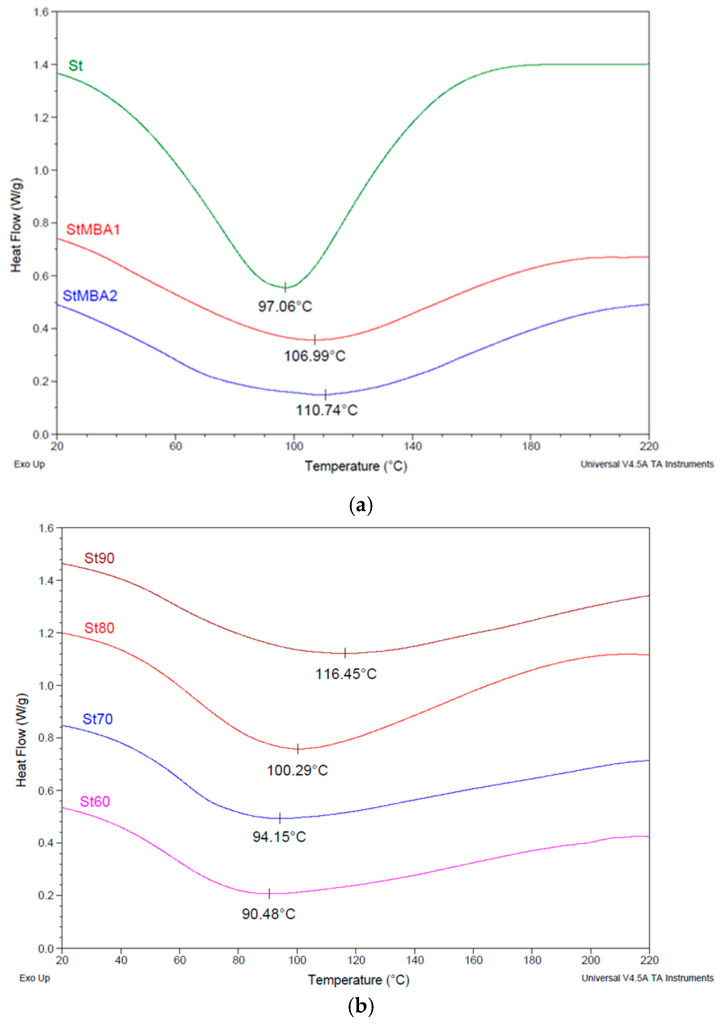
Differential scanning calorimetry: (**a**) of native potato starch (St) and cross-linked starches (StMBA1 and StMBA2); (**b**) cross-linked starch-g-polyacrylamide absorbents with different amount of natural polymer St90, St80, St70 and St60.

Figure 4 displays the DSC curves of starch (gelatinized St) and cross-linked starches (StMBA1 and StMBA2)—Figure 4a and cross-linked starch-g-polyacrylamide absorbents with different amount of natural polymer from 60 wt.% (St60) to 90 wt.% (St90)—Figure 4b. As know from the literature [42,45], starch is a semi-crystalline material. It swells when heated and an endothermic process is noted, therefore all DSC curves of the samples tested showed a similar trend—only one endothermic peak appeared.

For St, the peak gelatinization temperature—T_p_ was 97.06 °C, which is a relatively high temperature compared to the study by Hu et al. [42], which may be due to the nature of potato starch. However, cross-linked MBA starches (StMBA1 and StMBA2, Figure 4a) show higher temperatures for the endothermic peaks than St (106.99 °C and 110.74 °C respectively). In this case, the introduction of the hydrophilic amide groups in cross-linking agent MBA can be explained by the sub-linking of the material or a reduction of the free water [45].

On the other hand, the melting temperature—T_m_—was determined for starch SAPs with PAM [46], as the PAM content in the material increases, a shift of endothermic peaks on DSC curves toward lower temperatures is visible—Figure 4b. The peak temperature T_m_ for St90 was 116.45 °C, for St80 was 100.29 °C, for St70—94.15 °C and for St60 was 90.48 °C. In this case, the incorporation of the hydrophilic amide groups with PAM reduce the characteristic temperature of starch SAPs.

#### 3.2.4. TGA Characterization of Starch SAPs

**Figure 5:**
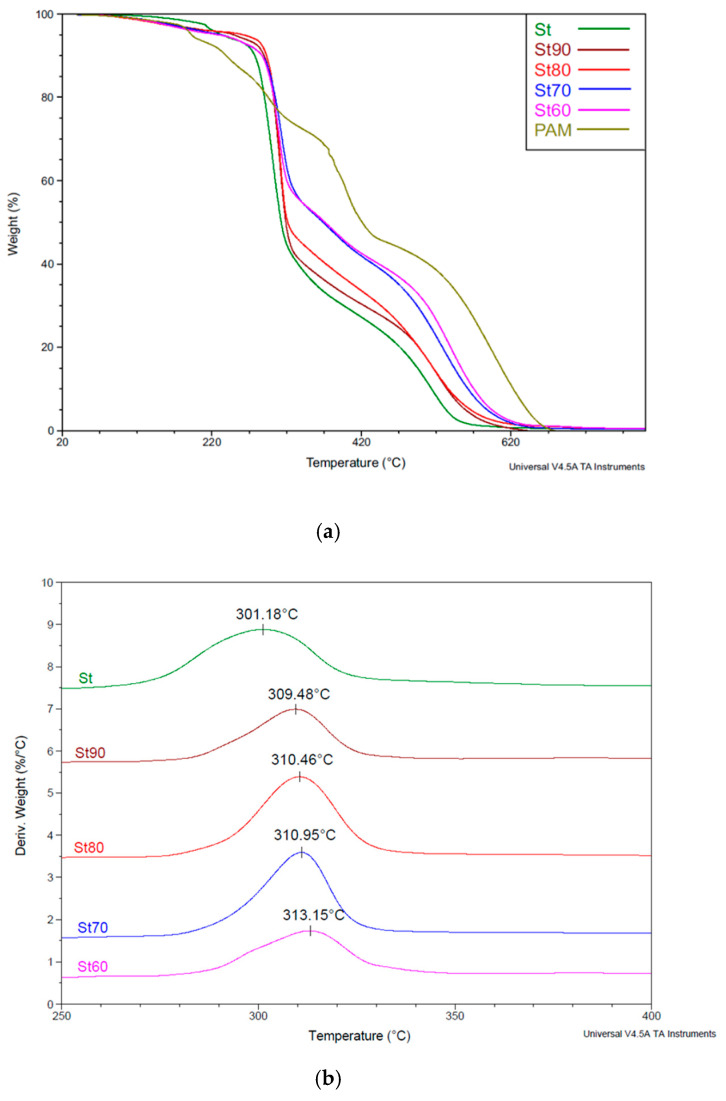
The thermogravimetric curves (**a**) St, polyacrylamide-PAM, St90, St80, St70 and St60; (**b**) DTG curves of starch St and starch SAPs.

The results of the thermogravimetric studies are shown in Figure 5, the TGA profiles of native potato starches and their corresponding starch SAPs are present in Figure 5a (with an additional TGA profile for pure PAM for comparison purposes) and the DTG for starch SAPs (Figure 5b).

Potato starch showed significant and fastest weight loss at 301.18 °C (Figure 5). According to the literature [30,38], the decomposition of the starch SAPs shows three unambiguous stages (Figure 5a). These are (i) the dehydration at 30–220 °C, (ii) the degradation of starch at 250–350 °C, and (iii) the degradation of PAM chains at 380–620 °C. As the PAM content in the starch SAPs increases from St90 to St60, the decomposition temperature shifts toward higher temperatures. The fastest mass losses for individual SAPs were recorded at temperatures corresponding to the following order: St—301.18 °C < St90—309.48 °C < St80—310.46 °C < St70—310.95 °C < St60—313.15 °C (Figure 5b). The higher is PAM content in starch SAPs, the stronger is the interaction between the starch chains and PAM.

#### 3.2.5. X-ray Diffraction (XRD)

**Figure 6:**
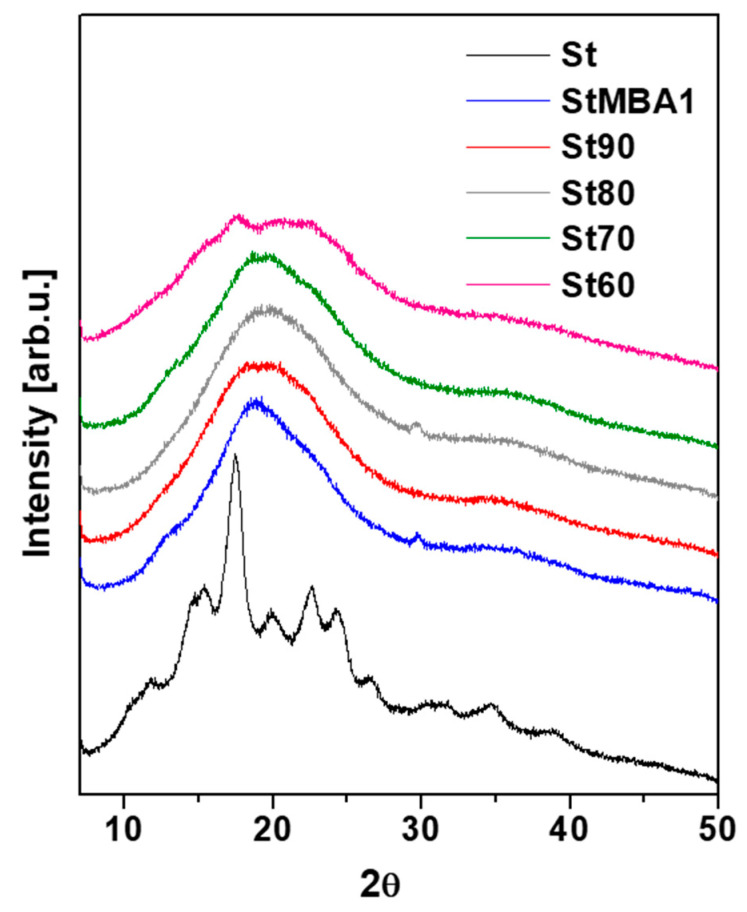
Wide angle X-ray powder diffraction spectra for potato starch, crosslinked starch and starch SAPs.

The XRD patterns of native potato starch, StMBA1 and all starch-g-polyacrylamide SAPs were determined and shown in Figure 6. The St gave a strong diffraction peak at about 17.5° 2θ, three weak peaks at around 2θ values of 20°, 22° and 25°, and an additional peak at 15° 2θ, which is the characteristic scattering features of B-type crystal from the retrograded starch chains [29,47,48]. The type of XRD pattern changed in the case of the SAP samples and the crystal peaks almost disappeared. The samples: StMBA1, St90, St80 and 70 were nearly amorphous with a large halo in the spectrum. This shows the destruction of crystalline structure of starch during the grafting and crosslinking reaction. Only sample with the highest PAM content (St60) showed small peak corresponding to the crystallinity of grafted PAM chain on starch.

### 3.3. Adsorption from Cations Solution

#### 3.3.1. Adsorption of Cu(II)

**Figure 7:**
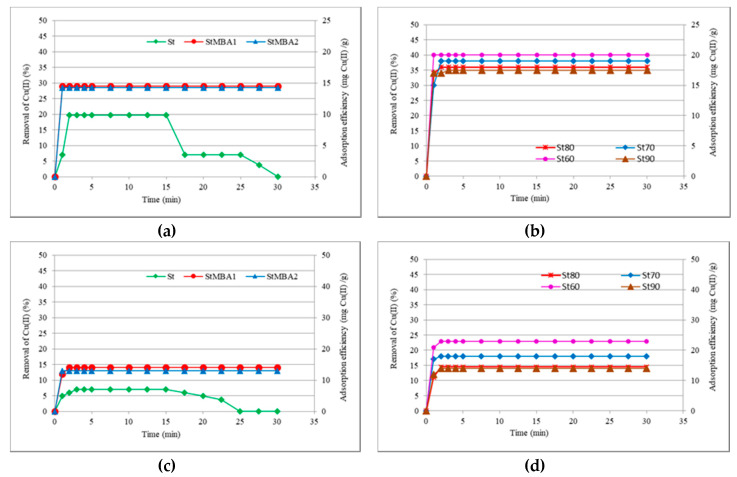
The removal of Cu(II) (%) and adsorption efficiency (mg Cu(II)/g) in time of initial concentration 50 Cu(II) mg/100 cm^3^ (**a**) for St, StMBA1 and StMBA2; (**b**) for starch SAPs with different amount of natural polymer; for initial concentration 100 Cu(II) mg/100 cm^3^ (**c**) for St, StMBA1 and StMBA2; (**d**) and for starch SAPs.

Heavy metal adsorption from the relatively high single metal aqueous solutions (50 and 100 mg Cu(II) mg/100 cm^3^) was studied in the adsorption experiments. The effect of treatment time, initial metal ion concentration and composition of starch graft copolymers on the adsorption of Cu(II) were considered (Figure 7, Table 1).

Figure 7a,c illustrate the effect of MBA content on the cooper(II) adsorption efficiency of cross-linked starch and potato starch. The removal of Cu(II) from solution increases with the presence of the cross-linking agent in both cases. The adsorption efficiency for StMBA1 and StMBA2 is 14 and 13 mg Cu(II), respectively, per gram of sorbent, regardless of the initial concentration of Cu(II) ions. However, the corresponding removal of Cu(II) decreased twice from 28.5% to 14% for StMBA1 (0.5 wt.%)—inversely proportional to the initial concentration of Cu(II) ions in solution. The StMBA2 with a higher MBA content (1 wt.%) shows slightly worse sorption properties of Cu(II) than StMBA1. Overall, more MBA content increased the cross-link density, thereby reducing the distance between adjacent intersections. This generates a tighter network and makes it difficult for metal ions to penetrate and adsorb [42]. Adsorption efficiency of Cu(II) for StMBA (1 and 2) after 24 h was above 7.0 mg Cu(II)/g and decreased to 3.9 mg/g for the initial concentration of 50 mg Cu(II) mg/100 cm^3^, while for the concentration of 100 mg Cu(II) mg/100 cm^3^, even after 2 days the adsorption efficiency remained at the level of 7.1–7.2 mg Cu(II)/g (Table 1). Purification of the solution from copper ions for St was worse than for starch with MBA and, importantly, not stable over time. The entire solution becomes cloudy and the starch grains do not settle.

Figure 7b,d illustrate the effect of starch graft copolymers composition and initial metal ion concentration on the adsorption of Cu(II). Regardless of the concentration of the starting copper(II) solution, the removal of Cu(II) from the solution is more effective with an increase in the proportion of PAM in the starch SAP. The most satisfactory purification effects was obtained for St60 and it was 40% for the lower concentration of copper (Figure 7b) and 23% for the larger concentration (Figure 7d). The lowest removal of metal ions among starch SAPs was obtained for St90 and it was 35% (Figure 7b) and 14% (Figure 7d), respectively. All the synthesized cross-linked starch-g-PAMs showed better sorption properties than the cross-linked starch. The increase of the adsorption efficiency for SAPs compared with the StMBA could be attributed to the higher complex formation rate between Cu(II) ions and the amine groups on the surface of the starch graft copolymers [49]. The adsorption efficiency for SAPs is ordered: St90—17.5 mg/g < St80—18 mg/g < St70—19 mg/g < St60—20 mg/g (Figure 7b) and St90—14 mg/g < St80—15 mg/g < St90—18 mg/g < St60—23 mg/g (Figure 7d).

Adsorption efficiency of Cu(II) for starch SAPs after 1 and 2 days of treatment decreased slightly e.g., to 19.5 mg/g—St60 for the initial concentration of 50 mg Cu(II) after 24 h or to 20 mg/g—St60 for the initial concentration of 100 mg Cu(II) after 48 h (Table 1). The elongation of Cu(II) sorption tests time to 48 h, showed weakening of the sorption properties of starch SAPs with time.

#### 3.3.2. Adsorption of Fe(III)

**Figure 8:**
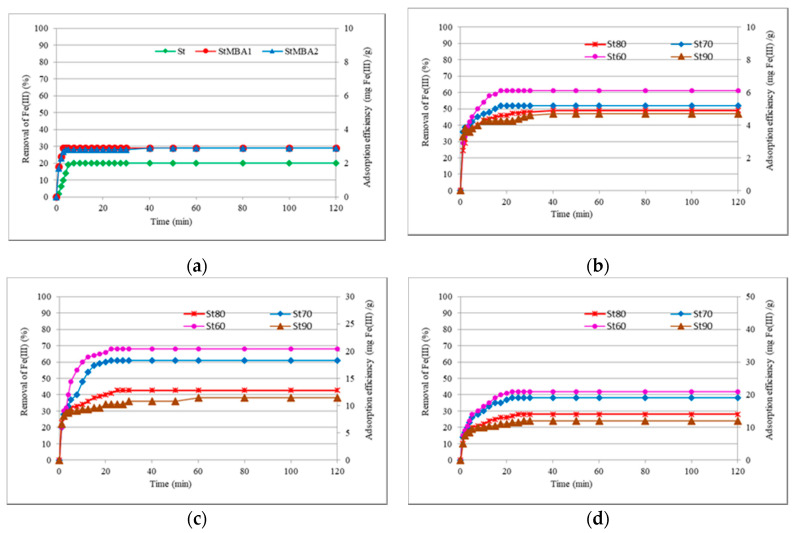
The removal of Fe(III) (%) and adsorption efficiency (mg Fe(III)/g) in time of initial concentration 10 Fe(III) mg/L (**a**) for St, StMBA1 and StMBA2; (**b**) for starch SAPs with different amount of natural polymer; (**c**) for starch SAPs of initial concentration 30 Fe(III) mg/L; (**d**) for starch SAPs of initial concentration 50 Fe(III) mg/L.

The adsorption of Fe(III) from the aqueous solutions (10, 30 and 50 mg Fe(III)/L) was also investigated. The results are included in Figure 8 and Table 2.

Figure 8a visualizes the effect of MBA content on the removal of iron(III) and adsorption efficiency for StMBAs and St. The removal of Fe(III) from solution (10 Fe(III) mg/L) for StMBA1 and StMBA2 is almost equal—29% and adsorption efficiency is 2.9 mg/g. The similarity in sorption properties for both cross-linked MBA starches was observed for all solutions with different Fe(III) concentrations both after 24 and 48 h of experiment duration (Table 2). The absorption efficiency of Fe(III) mg/g for StMBAs was in a range between 4.0 and 5.5 mg/g after 48 h and increased with an increase in the initial concentration of iron cation. St itself also showed slight sorption properties of Fe(III) in the range of 2.0–2.5 mg/g (Figure 8a and Table 2).

The removal of Fe(III) ions from solutions (Figure 8b–d), both during the first 2 h of tests and after 24 or 48 h, significantly increases with the increase of the amount of PAM in the starch copolymer (Table 2). Even a slight change of StMBA1 in ST90 copolymer significantly improves the adsorption efficiency with 1.6 multiplicity for the concentration of Fe(III) 10 mg/L (4.7 mg/g—Figure 8b), almost four times for Fe(III) 30 mg/L (11.4 mg/g—Figure 8c) and more than four times for Fe(III) 50 mg/L (12 mg/g—Figure 8d). The best results were achieved for the starch SAP with the highest PAM content—St60. The removal of iron cation by St60 was 61% (Figure 8b), 68% (Figure 8c) and 42% (Figure 8d). It is worth emphasizing that the removal level of Fe(III) from the solutions lasted up to 24 h and remained at the same level for another day; 75% for the concentration of Fe(III) 10 mg/L, 72% for the concentration of 30 and 44% for the 50 mg/L (Table 2). It proves the strong binding of the Fe(III) cation by the SAPs obtained by the described method.

The Fe(III) adsorption efficiency is strongly correlated with the composition of starch-g-polyacrylamide SAP. This phenomenon can be explained by the presence of amide groups (–CONH_2_) in polymer materials that attracted Fe(III) ions by complexing ability [28]. Additionally, gelatinized polysaccharides form high-spin complexes with many metal cations including Fe(III) and could form complexes either by a sorption of Fe(III) on the surface or by penetration of metal ions into materials [50].

The results of the adsorption efficiency have not been found in the available literature for identical materials as those discussed in the presented manuscript, therefore Table 3 summarizes AE values for similar adsorbents. The adsorption efficiencies for starch-g-PAM were mostly greater than most of the other adsorbents previously reported in the literature.

#### 3.3.3. Pseudo-Second Order Model

**Figure 9:**
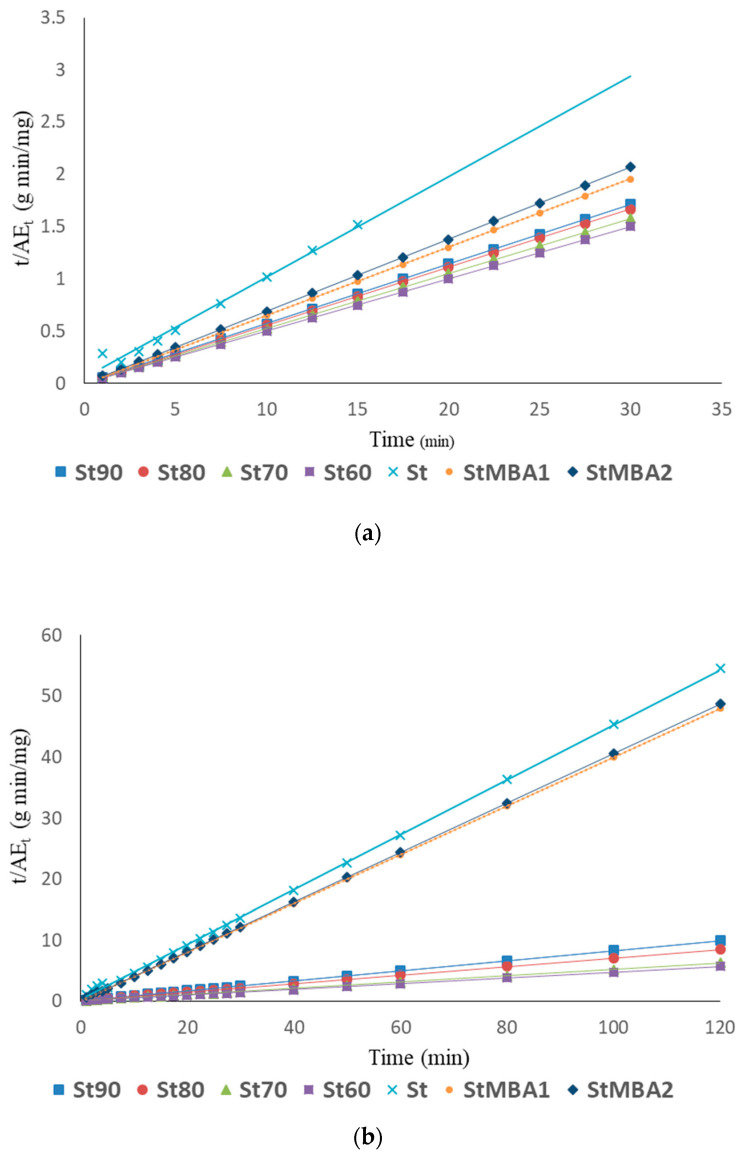
Second order plots for St, StMBA1, StMBA2 and starch SAPs with different amount of natural polymer (**a**) for Cu(II)—initial concentration 50 Cu(II) mg/100 cm^3^; (**b**) for Fe(III)—initial concentration 50 Fe(III) mg/L.

Study of chemical kinetics includes careful monitoring of the experimental conditions which influence the speed of a chemical reaction and hence, help attain equilibrium in a reasonable length of time. Such studies yield information about the possible mechanism of adsorption on the way to the formation of the final adsorbate–adsorbent complex and help develop appropriate mathematical models to describe the interactions. Applications of second order kinetic models of adsorption systems it is recommended for research on biomaterials [56]. Due to the similarity of all obtained second order plots of adsorption, the ones selected for copper(II) and iron(III) are shown. Figure 9 displays the second order plots for Cu(II) adsorbed on St, StMBA1, StMBA2 and starch SAPs with different amount of natural polymer of initial concentration 50 Cu(II) mg/100 cm^3^ (Figure 9a) and for Fe(III) of initial concentration 50 Fe(III) mg/L (Figure 9b) However, the k coefficient was determined and tabulated for all tested systems (Table 4).

**Scheme 3:**
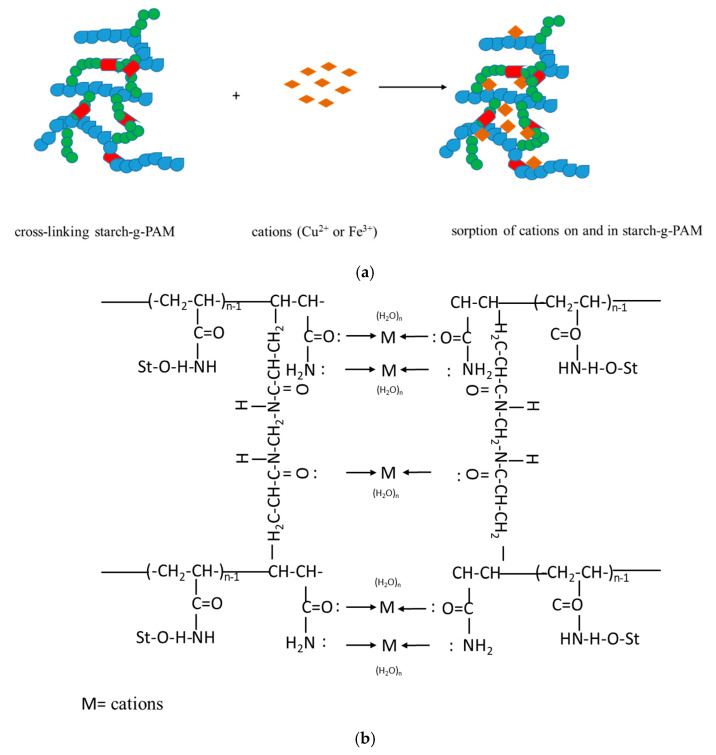
The schematic illustration of: (**a**) hydrogel fabrication; (**b**) adsorption mechanism.

According to the theoretical investigations and the experimental studies extensively described by Gupta et al. [41] that the value of k usually depends on the initial adsorbate concentration in the bulk phase. The rate coefficient, k decreases with the increasing initial adsorbate concentration as a rule. The described relationship for k is demonstrated for St, StMBA1 and StMBA2 (in bold in Table 4). This confirms that in these cases the decisive mechanism of metal adsorption is on the surface of the absorbents and according to the second order kinetic model is most likely to involve chemical interactions leading to binding of the ions to the surface by bonding as strong as e.g., covalent bonding. On the other hand, all SAPs based on cross-linked starch copolymers do not show a clear dependence of the k coefficient on the initial concentration of ion solutions. The ion sorption mechanism is much more complex. In addition to the chemical adsorption of ions on the surface, there is sorption inside the polymer network and chelating mechanism may generally dominate. The schematic illustration of hydrogel fabrication and a mechanism of metals adsorption is suggested in Scheme 3 (a and b respectively). The proposed adsorption scheme is shown the probable co-ordinate bond between the donor atoms in the sites where modified starch absorbents bind to metal ions. The quality of the network formed in the polymer and its ability to swell (see Section 3.3.4) and thus to penetrate and retain ions inside the material have a decisive influence on the sorption of cations.

#### 3.3.4. Swelling in Cation Solution

**Figure 10:**
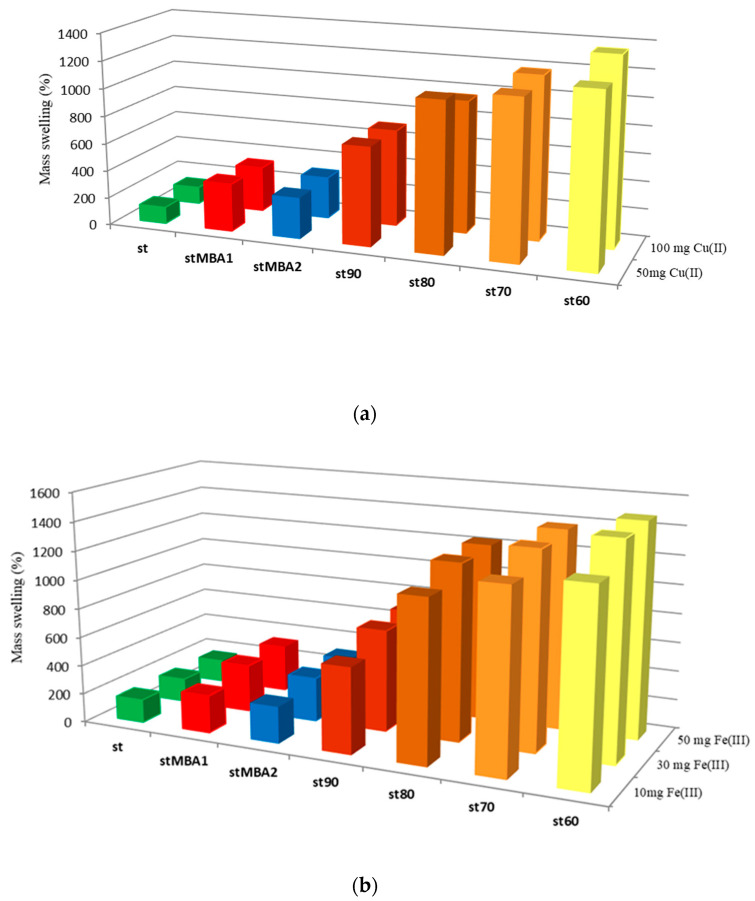
Mass swelling of potato starch (St) and cross-linked starches (StMBA1 and StMBA2) and cross-linked starch-g-polyacrylamide absorbents with different amount of natural polymer St90, St80, St70, St60 after 48 h in (**a**) Cu(II) solutions; (**b**) Fe(III) solutions.

The swelling properties of all samples were tested simultaneously with adsorption efficiency of ion tests. Definitely, the swelling capacity of the obtained materials strongly depends on the composition of SAPs and correlates very well with the adsorption properties. Cumulative swelling results for starch materials are presented in Figure 10, where Figure 10a shows the results for Cu(II) solutions, while Figure 10b for Fe(III) solutions; Equation (5) was used for mass swelling calculations. The mass swelling of St was the smallest and comparable in all metal cation solutions used. The cross-linked StMBA starches achieved comparable weight increase (2.5 to 3.2 fold) in all solutions. The swollen starch-g-polyacrylamide SAPs were homogenous gels with a delicate color derived from the appropriate cation. The increase in swelling of the materials coincides with the increase in the proportion of PAM content in the absorbent from St90 to St60.

The largest amount of cation solution was absorbed by St60 (12–13 times in Cu(II) solutions and 12–14.6 times in Fe(III) solutions) compared to St. Changes in the initial concentration of the starting metal cation solutions had a smaller influence on the swelling properties of starch materials than the structure of copolymers. Even a small amount of grafted PAM in the material made visible increase in St90 swelling. These excellent swelling properties may be due to the hydrophilicity of polyacrylamide, adequate grafting of PAM onto starch, and a well-chosen amount of cross-linker. The change in the structure of the tested materials is also important for the swelling properties (see Figure 3).

## 4. Conclusions

Cross-linked potato starches and starch grafted polyacrylamide copolymers were prepared by chemical crosslinking polymerization using MBA. The starch SAPs were synthesized by free radical initiated polymerization with ammonium persulfate as initiator in double chemical-chemical modification (grafting and cross-linking). The characteristics of the obtained materials confirmed their diversity in terms of composition and structure. Their adsorption behavior toward copper(II) and iron(III) ions from aqueous solutions was investigated under various conditions such as treatment time, relatively high concentration of metal ions, content of MBA on cross-linked starch as well as content of grafted PAM on starch chains. It was found that cross-linked starches can adsorb metal ions; however, increasing the amount of cross-linking agent does not improve sorption properties. The optimal dose of MBA turned out to be the concentration of 0.5 wt.%. It has been proved that starch-g-PAM SAPs have much better Cu(II) and Fe(III) adsorption properties than StMBAs and the ability of SAPs to adsorb metals increases with increasing grafting of polyacrylamide onto starch chains. The adsorption efficiency for SAPs is ordered: St90 < St80 < St70 < St60 and removal of cations depends on initial solution concentration. The best results of solutions purification from Cu(II) was 23 mg/g and from Fe(III)—21.2 mg/g for St60. However, the Cu(II) adsorption efficiency after 1 and 2 days of treatment slightly decreased. For all tested materials, absorbed amount of iron cations was persisted even after 48 h. The obtained results are mostly better than the results for similar materials presented so far in the literature. The adsorption properties of the tested materials are significantly correlated with the presented mathematical model and with the swelling capacity. Satisfactory adsorption properties ware explained by the proposed cations sorption mechanism, the ability to form complexes with metal cations, adequate grafting of PAM onto starch, a well-chosen amount of cross-linker as well as changes in the material structure.

## Figures and Tables

**Table 1:** The removal of Cu(II) (%) and adsorption efficiency [mg Cu(II)/g] after 24 and 48 h from two initial concentration of Cu(II) for St, StMBAs and starch SAPs with different amount of PAM.

	Removal (%)		Absorption Efficiency (mg/g)		Removal (%)		Absorption Efficiency (mg/g)	
Concentration (mg/100 cm^3^)	50				100			
Time (h)	24	48	24	48	24	48	24	48
St	0.0	0.0	0.0	0.0	0.0	0.0	0.0	0.0
StMBA1	15.4	7.8	7.7	3.9	8.4	7.2	8.4	7.2
StMBA2	14.5	7.6	7.2	3.8	7.6	7.1	7.6	7.1
St90	35.0	30.0	17.5	15.0	11.0	10.8	11.0	10.8
St80	35.0	30.2	17.5	15.1	11.0	10.8	11.0	10.8
St70	38.0	30.0	19.0	15.0	17.2	15.9	17.2	15.9
St60	39.0	30.0	19.5	15.0	21.0	20.0	21.0	20.0

**Table 2:** The removal of Fe(III) (%) and adsorption efficiency (mg Fe(III)/g) after 24 and 48 h for three initial concentration of Fe(III) 10, 30 and 50 mg/L for St, StMBA1, StMBA2 and starch SAPs with different amount of PAM.

	Removal of Fe(III) (%)		Absorption Efficiency of Fe(III) (mg/g)	
**Time (h)**	24	48	24	48
**Concentration of Fe(III) (mg/L)**	10			
St	20	22	2.0	2.2
StMBA1	34	40	3.4	4.0
StMBA2	32	40	3.2	4.0
St90	52	52	5.2	5.2
St80	61	61	6.1	6.1
St70	68	70	6.8	7.0
St60	75	75	7.5	7.5
**Concentration of Fe(III) (mg/L)**	30			
St	8.0	8.0	2.4	2.4
StMBA1	15	16	4.5	4.8
StMBA2	15	15	4.5	4.5
St90	50	50	15.0	15.0
St80	52	51	15.6	15.3
St70	65	65	19.5	19.5
St60	72	72	21.6	21.6
**Concentration of Fe(III) (mg/L)**	50			
St	5.0	5.0	2.5	2.5
StMBA1	10	11	5.0	5.5
StMBA2	9	9	4.5	4.5
St90	25	25	12.5	12.5
St80	29	30	14.5	15.0
St70	40	39	20	19.5
St60	44	44	22	22

**Table 3:** Adsorption efficiencies (AE) of Cu(II) and Fe(III) for various adsorbents with maximum AE.

Ion	AE (mg/g)	Adsorbent	Comments	Reference
Cu(II)	24.05	Poly(acrylamide-co-sodium methacrylate)	lack of starch	[51]
	-	Starch-g-polyacrylamide		[25]
	20.33	Dithiocarbamate-modifed glycidyl methacrylate starch	lack of PAM	[52]
	40.72	polyacrylamide with anionically modified potato starch	theoretical sorption, 12.28 wt.% of starch	[27]
	23.0	Starch-g-polyacrylamide	60–90 wt.% of starch	This work
Fe(III)	12.9	nano-hydroxyapatite (n-HAp) with chitin and chitosan	lack of starch and PAM	[53]
	20.9	clinoptilolite	lack of starch and PAM	[54]
	13.6	hazelnut hull	lack of starch and PAM	[55]
	21.2	Starch-g-polyacrylamide		This work

**Table 4:** The second order rate coefficient “k” from pseudo-second order mathematical model (determined from the slope and intercept of a plot of t/AE_t_ against t) of St, StMBA1, StMBA2 and starch SAPs with different amount of natural polymer for Cu(II) and Fe(III) adsorption from different initial concentration of ions solutions.

Ion	Cu(II)		Fe(III)		
**Initial Concentration** ^1^	**50**	**100**	**10**	**30**	**50**
St	**0.5890**	**0.2186**	**1.6971**	**0.9706**	**0.7902**
StMBA1	**0.0046**	**0.0005**	**0.5446**	**0.2109**	**0.1114**
StMBA2	**0.0039**	**0.0028**	**0.5668**	**0.2360**	**0.1947**
St90	0.0158	0.0323	1.3330	2.2934	1.5104
St80	0.0108	0.0610	1.3050	1.5110	1.7124
St70	0.0512	0.0108	0.7060	2.0338	1.8482
St60	0.0004	0.0184	1.0290	1.6576	2.0086

^1^ Unit of concentration for Cu(II)—(mg/100 cm^3^) and for Fe(III) (mg/L).
